# Identification of a small molecule inhibitor of Ebola virus genome replication and transcription using *in silico* screening

**DOI:** 10.1016/j.antiviral.2018.06.003

**Published:** 2018-08

**Authors:** Victoria Easton, Martin McPhillie, Isabel Garcia-Dorival, John N. Barr, Thomas A. Edwards, Richard Foster, Colin Fishwick, Mark Harris

**Affiliations:** a School of Molecular and Cellular Biology, Faculty of Biological Sciences, University of Leeds, Leeds LS2 9JT, UK; b School of Chemistry, Faculty of Mathematics and Physical Sciences, University of Leeds, Leeds LS2 9JT, UK; c Astbury Centre for Structural Molecular Biology, University of Leeds, Leeds LS2 9JT, UK; d Institute of Infection and Global Health, University of Liverpool, Liverpool L3 5RF, UK

**Keywords:** Ebola virus (EBOV), Nucleoprotein (NP), Small molecule inhibitors (SMIs), Minigenome, Drug discovery

## Abstract

Ebola virus (EBOV) causes a severe haemorrhagic fever in humans and has a mortality rate over 50%. With no licensed drug treatments available, EBOV poses a significant threat. Investigations into possible therapeutics have been severely hampered by the classification of EBOV as a BSL4 pathogen. Here, we describe a drug discovery pathway combining *in silico* screening of compounds predicted to bind to a hydrophobic pocket on the nucleoprotein (NP); with a robust and rapid EBOV minigenome assay for inhibitor validation at BSL2. One compound (MCCB4) was efficacious (EC_50_ 4.8 μM), exhibited low cytotoxicity (CC_50_ > 100 μM) and was specific, with no effect on either a T7 RNA polymerase driven firefly luciferase or a Bunyamwera virus minigenome. Further investigations revealed that this small molecule inhibitor was able to outcompete established replication complexes, an essential aspect for a potential EBOV treatment.

## Highlights

• An EBOV drug discovery pathway which is performed at BSL2 and successfully identifies SMIs.
• MCCB4 is a SMI of EBOV which is effective, specific and not cytotoxic.
• The effect of MCCB4 was demonstrated in two cell types.
• MCCB4 is able to outcompete established EBOV replication complexes.
• SAR analysis was performed with 2nd generation compounds.

## 1 Introduction

The *Ebolavirus* genus, alongside *Cuevavirus* and *Marburgvirus,* is classified within the *Filoviridae* family within the order *Mononegavirales*. There are 5 species within the genus: *Bundibugyo* (BDBV), *Reston* (RESTV), *Sudan* (SUDV), *Taï Forest* (TAFV) and *Zaire* (previously known as ZEBOV) the type species now referred to as *Ebola virus* (EBOV) (Amarasinghe et al., 2017; Kuhn et al., 2010). Marked differences can be seen between the different species with regard to geographical spread and pathogenicity. For example EBOV can exhibit disease mortality rates of up to 90% in humans (Rollin, 2009), while RESTV is not known to cause disease in humans (Miranda and Miranda, 2011).

The high pathogenicity of EBOV, the ease of transmission via bodily fluids (Bausch et al., 2007), the rapid infection progression (CDC, 2014), and the current lack of licenced treatments has resulted in its classification as a Biosafety Level 4 (BSL4) pathogen, hampering development of effective therapies. Hence, despite much research on EBOV replication and potential therapeutics there are currently no licenced treatments for infection.

EBOV is a filamentous enveloped virus with a non-segmented, negative sense single stranded RNA (-ssRNA) genome of ∼19 kb (Geisbert and Jahrling, 1995; Kiley et al., 1982). The genome encodes 7 proteins: a nucleoprotein (NP), a glycoprotein, 4 viral proteins (VP24, VP30, VP35 and VP40) and the L protein (RNA-dependent RNA-polymerase) (Mühlberger et al., 1999).

The NP forms a complex with VP35, VP30, and L which is essential for genome replication and transcription (Ruigrok et al., 2011; Sun et al., 2012; Zhou et al., 2013). This complex is the basis for the EBOV minigenome system (MG) (Mühlberger et al., 1999) where plasmids expressing these 4 proteins under the control of a T7 promoter are transfected into cells constitutively expressing T7 RNA polymerase, together with a plasmid with a T7 promoter driving production of an RNA containing the reverse complement of a reporter gene (firefly luciferase) flanked by EBOV genome recognition sequences. A functional replication complex will recognise these sequences, transcribe the reporter and allow translation of luciferase which provides an indirect measurement of EBOV-specific gene expression. Because the complete genome is not present and therefore no infectious virus can be produced, this system allows for the investigation of EBOV genome replication and transcription at BSL2.

Recently, the structure of the NP and the interactions with VP35 have been characterised (Dong et al., 2015; Leung et al., 2015). A hydrophobic pocket on NP either binds intramolecularly with a flexible arm of NP (helix-20), or with an NP binding peptide of VP35 (NPBP, residues 20–48). The two binding states control the binding of NP and release of RNA and oligomerisation – essential to viral replication (Kirchdoerfer et al., 2015). For other negative-strand viruses, it has been shown that NP is a valid target for small molecule inhibitors (SMIs), exemplified by the influenza inhibitor Nucleozin, which triggers aggregation of NP with an EC_50_ in the nM range (Kao et al., 2010), and the <60 nM EC_50_ reported for a series of inhibitors which promote NP oligomerisation (Gerritz et al., 2011). Another reason why NP is an attractive target for possible inhibitors is the VP35 binding pocket is highly conserved between EBOV and the related *Marburgviruses* (Zhu et al., 2017). Although VP35 NPBPs bind with a stronger affinity to their own NPs, they are able to bind to the NP of other filoviruses.

Although the MG system has been used recently to identify small molecule inhibitors of EBOV replication (Edwards et al., 2015; Luthra et al., 2018; Nelson et al., 2017; Welch et al., 2016), these studies have involved high throughput screens of pre-existing libraries of known bioactive compounds. We sought to refine this approach by combining it with a virtual screening cascade to identify compounds - available within our in-house chemical libraries - predicted to bind to the NP pocket. This combination identified a range of small molecule inhibitors of EBOV genome replication, one of which (MCCB4) is described here. The predicted binding was validated using an EBOV MG assay and further investigated at a variety of time points, in multiple cell lines, by binding site mutation and through structure-activity relationship (SAR) analysis.

## 2 Materials and methods

### 2.1 Cell lines and plasmids

BSR-T7 cells are derived from BHK-21 cells and stably express T7 RNA polymerase (Buchholz et al., 1999). Huh7-Lunet-T7 cells are a derivative of Huh7 cells and also express T7 RNA polymerase (Backes et al., 2010). Cells were grown in DMEM (Sigma-Aldrich), 10% FBS (Sigma-Aldrich), 1% non-essential amino acids (Lonza), penicillin-streptomycin (100 units/mL) (Sigma) with either G418 (600 μg/ml) (Life Technologies) (BSR-T7) or Zeocin (Invitrogen) (5 μg/ml) (Huh7-Lunet-T7) added at every second passage. Cells were maintained at 37 °C with 5% CO_2_.

The minigenome system for EBOV (Makona strain, GenBank KJ660347.2) (García-Dorival et al., 2016) expressing Firefly luciferase, used in this study was kindly donated by Julian Hiscox (University of Liverpool) following the minigenome system model developed by Mühlberger et al. (1999) (see Supplementary Fig. 1). The control plasmid pT7FFLuc contains a Firefly luciferase under the direct control of a T7 promoter. The plasmids for the Bunyamwera minigenome express a *Renilla* luciferase and were described previously (Barr et al., 2003; Walter et al., 2011).

### 2.2 Compounds

These were initially selected from our Medicinal Chemistry/Chemical Biology (MCCB) group in-house compound library in the School of Chemistry, University of Leeds. Compounds are stored at 14 °C in 100% DMSO stock solutions, within 0% humidity cabinets. Dry stocks of the original compounds were later purchased from the original supplier (ChemDiv) to confirm initial biological activity. Follow-up compounds were purchased from ChemBridge or ChemDiv and shipped as dry stocks. Compound purity was assessed by LCMS using standard methods and a cut-off of >95% purity was used.

### 2.3 Virtual screening cascade

The in-house library containing 48,750 small molecules was docked to both crystal structures of Ebola NP (4Z9P and 4YPI) using the exhaustive docking algorithm eHiTs (Zsoldos et al., 2007).

The small molecules were then ranked according to predicted binding affinity (eHiTs score). The top 5000 scoring molecules for each protein structure were re-docked using an alternative docking software, AutoDock 4.2 (Morris et al., 2009). The 5000 molecules were clustered by energy (predicted binding affinity) and an arbitrary cut-off of −7.5 was chosen for visually inspection. Since 20 conformations are produced for each structure, visual inspection allowed us to ascertain good clustering (>10/20 conformations were of similar energy, i.e. a highly populated pose), and identify predicted hydrogen bonds and steric clashes with the protein. Compounds that satisfied this criteria were cross-referenced with the top ranking molecules from eHiTs docking to identify those which were predicted to bind favourably in both crystal structures and both docking software. We envisioned that a consensus docking approach (and orthogonal scoring functions) with both crystal structures would increase our hit-finding. Additional *in silico* analysis was performed using Maestro 10.2 (Maestro, Schrödinger, LLC, New York, NY, 2017).

### 2.4 EBOV and BUNV minigenome and pT7FFLuc transfections

1 × 10^5^ cells were seeded in each well of a 24-well plate and allowed to adhere overnight to achieve a desired cell density of ∼90% confluency. Per well, cells were transfected with EBOV-MiniG 0.5 μg, NP 0.25 μg, VP30 0.125 μg, VP35 0.125 μg and L 0.125 μg; the pT7FFLuc 0.3 μg; or BUNV(S) REN 0.4 μg, L-sup 0.1 μg and S-sup 0.1 μg. Transfections were performed with Lipofectamine (ThermoFisher Scientific) according to manufacturer's instructions. Compounds for testing were made to the required concentrations in serum free DMEM directly before use and added to cells immediately before transfection. See Fig. 3B for deviations in compound addition.

### 2.5 Luciferase and MTT assays

Cells were harvested 24 hpt by washing in PBS then lysing in Passive Lysis Buffer (PLB, Promega) (See Fig. 3B legend for deviations in the timings of cell harvest). Samples were freeze/thawed at −20 °C and read using the Firefly or *Renilla* luciferase reporter assay systems (Promega) and a FLUOStar optima microplate reader.

MTT assays were performed alongside luciferase assays. After washing the cells in PBS, the MTT reagent (1 mg/mL in H_2_O) (Alfa Aesar) was added for 1 h. DMSO was used to lyse cells prior to being read on a Tecan Infinite F50 microplate reader. All plates contained a transfection only control and a cell only control to show 100% luciferase signal and background signal, respectively. 116 compounds were tested for effect against EBOV replication.

### 2.6 Site-Directed mutagenesis

Mutagenesis was performed on the NP plasmid, using a Q5 Site-Directed mutagenesis kit (NEB). Primers were designed using NEBaseChanger™ (sequences available on request) and contained silent *Acc*ll and *Sac*l restriction sites, respectively, for identification.

## 3 Results

### 3.1 *In silico* identification of small molecule inhibitors of NP-VP35 interaction

**Fig. 1:**
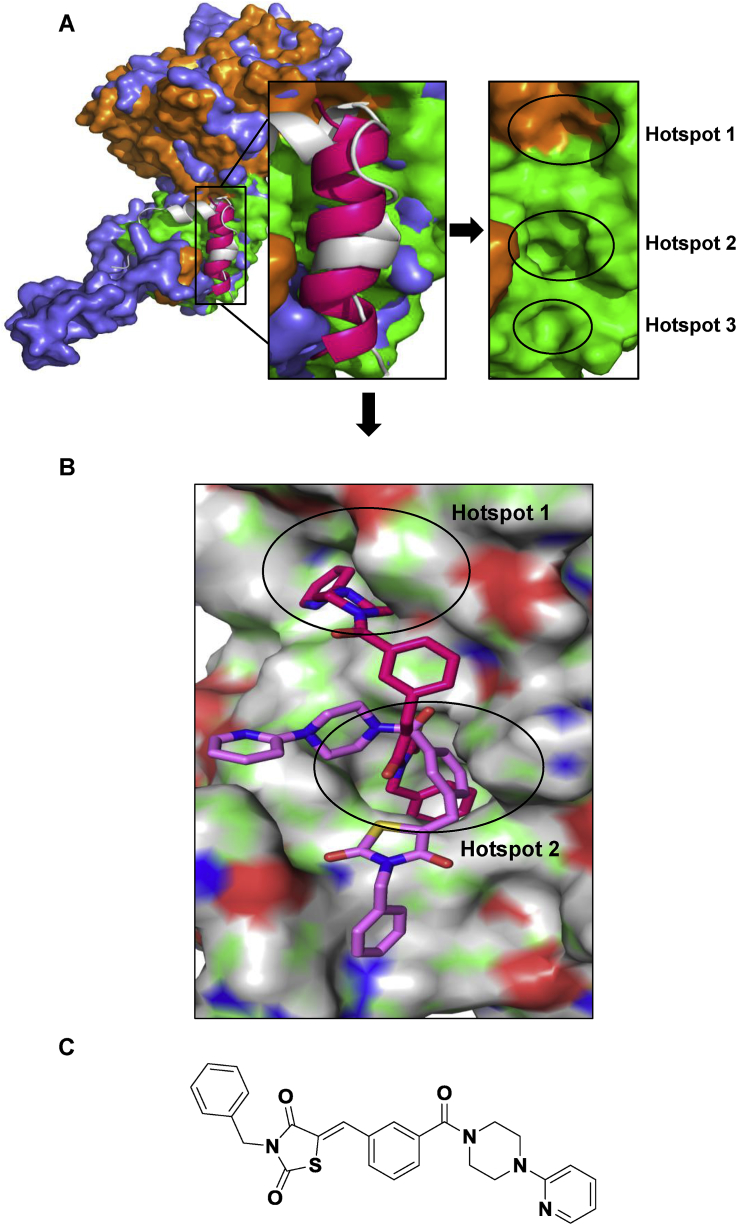
**Identification of MCCB4**. **A**. The EBOV NP structures - orange and green (Dong et al., 2015) and purple (Leung et al., 2015) - were merged to form the pocket where VP35 (white helix) or NP helix-20 (pink helix) bind. Three hydrophobic hotspots were identified as important for these protein:protein interactions. **B.** Compounds were virtually screened by eHiTs exhaustive docking engine (red structure) and AutoDock cluster analysis and pose prediction software (lilac structure). **C.** Structure of MCCB4. (For interpretation of the references to colour in this figure legend, the reader is referred to the Web version of this article.)

From 48,750 structures contained within the in-house compound library, 116 compounds were selected, based on the consensus scoring approach detailed above, for biological evaluation in the EBOV genome assay. Of these 11 compounds showed promising activity against EBOV at 100 μM. All hit compounds showed chemical diversity in heterocycles and functional groups, however, of the 11 active compounds, MCCB4 was chosen for further investigation as it had typical physicochemical properties: a molecular weight of 485 daltons, a calculated logP of 4.8, and contained no hydrogen-bond donors. It was predicted to bind over hotspots one and two (Fig. 1), in a similar manner to the other active compounds.

MCCB4 is a linear hydrophobic molecule containing an ene-thiazolidinedione group. This molecule has the potential to act as a Michael acceptor via covalent bond formation to nucleophilic residues within proteins, and display ‘promiscuous’ behaviour in biological assays (Baell and Walters, 2014). We were, therefore cautious concerning any biological activity observed for this molecule and wished in particular to probe the selectivity of the activity displayed by this molecule.

### 3.2 MCCB4 inhibits EBOV genome replication and transcription

In order to assess whether MCCB4 was able to inhibit EBOV genome replication and transcription, we utilised a minigenome (MG) system (Supplementary Fig. 1). As negative controls we also used a Bunyamwera virus (BUNV) MG system, and a plasmid in which FF-luc expression was directly driven by a T7 promoter. All EC_50_ and CC_50_ data curves demonstrated in this paper are shown as percentages of an in-plate DMSO only control. All data was generated from 3 independent experiments performed in triplicate, allowing us to minimise the effect of well-to-well variation in transfection efficiency.

**Fig. 2:**
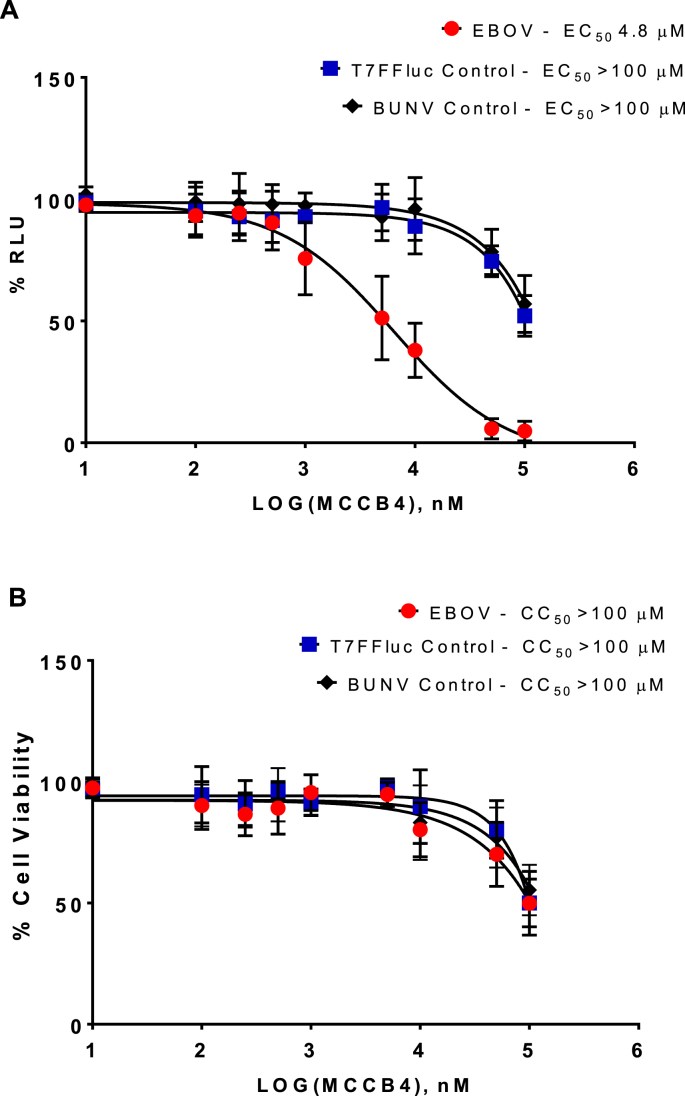
**Activity of MCCB4.** MCCB4 was tested for activity against the T7FFLuc control, the BUNV minigenome and EBOV minigenome. The compounds were added to BSR-T7 cells at 100, 50, 10, 5, 1, 0.5, 0.25 0.1 and 0.01 μM immediately before transfection. Cells were harvested at 24 hpt and analysed for Renilla or Firefly luciferase activity, or cell viability by MTT assay. Values are represented as percentages of a transfection only control. **A.** EC_50_ curve of percentage reduction in luciferase. EBOV, red circles. T7FFLuc control, blue squares. BUNV control, black diamonds. **B.** CC_50_ curve of percentage reduction in cell viability. EBOV, red circles. T7FFLuc control, blue squares. BUNV control, black diamonds. Averages plotted from 3 independent experiments performed in triplicate. Error bars show standard deviation (SD). (For interpretation of the references to colour in this figure legend, the reader is referred to the Web version of this article.)

Transfected cells were treated with MCCB4 over a 10^4^-fold range of concentrations from 10 nM to 100 μM. As shown in Fig. 2A, MCCB4 exhibited a dose-dependent inhibition of the EBOV MG with an EC_50_ of 4.8 μM. This was specific to EBOV as the EC_50_ for both BUNV MG and the T7FFLuc controls was >100 μM. In parallel, we confirmed that MCCB4 did not exhibit any significant toxicity (CC_50_ > 100 μM: Fig. 2B). This was assessed in cells transfected with the various combinations of plasmids to account for any additional toxicity due to expression of exogenous proteins (in particular those of EBOV).

**Fig. 3:**
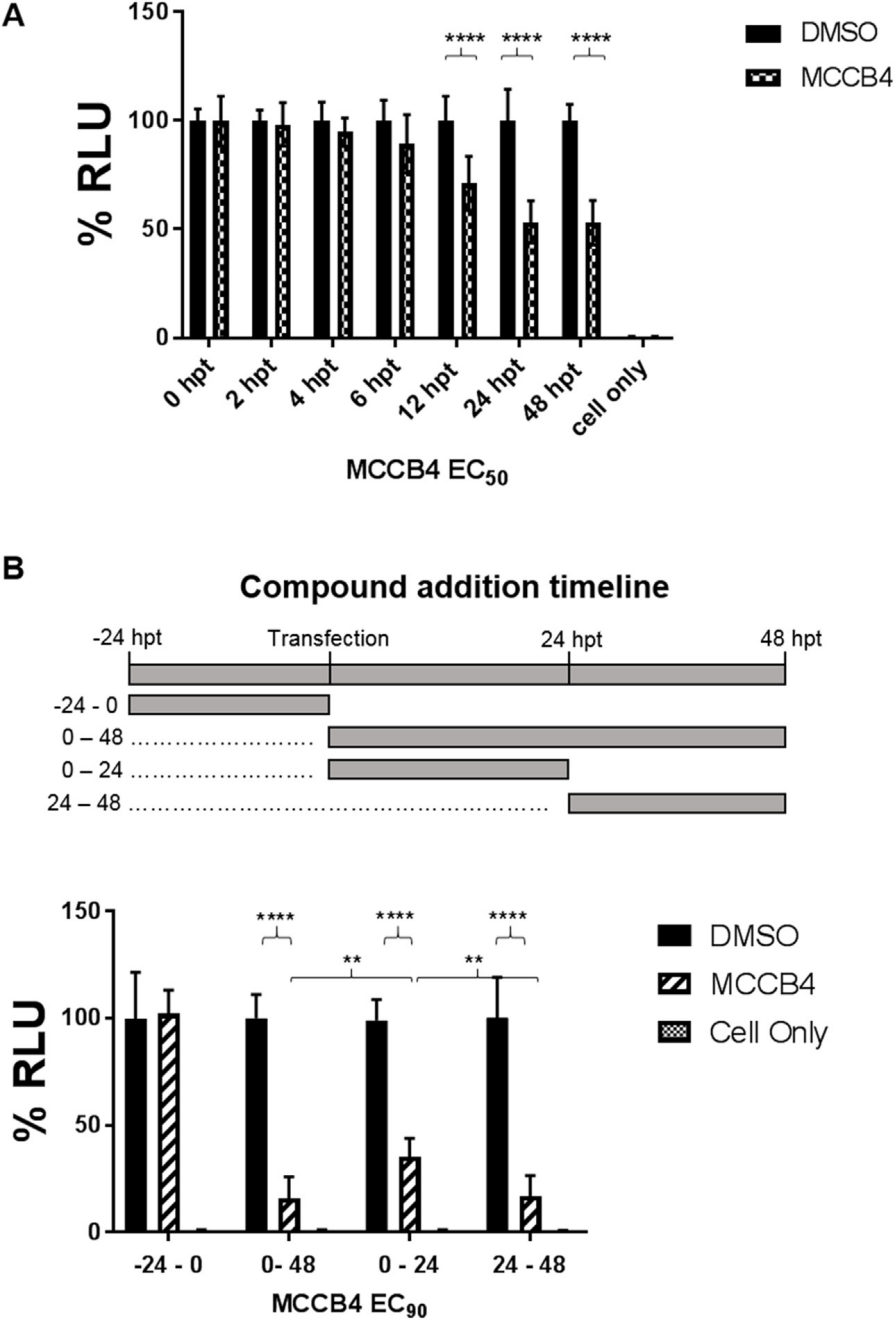
**Time-course analysis of the activity of MCCB4. A.** MCCB4 was analysed at the EC_50_. Samples were harvested at the indicated times hpt, values are represented as percentages of a transfection only control. Dashed line represents background of the assay. **B.** A time of addition study was undertaken at a concentration of MCCB4 equivalent to the EC_90_. The top panel is a schematic demonstrating when MCCB4 is present during transfection. The graph shows the data as a percentage of a transfection only DMSO control, harvested at the same time. All averages plotted from 3 independent experiments performed in triplicate and compared using an unpaired two-tailed *t*-test. Error bars show SD. **** P Value < 0.0001. ** P Value 0.01.

To characterise the behaviour of MCCB4 further, a time course was performed. BSR-T7 cells were transfected in the presence or absence of 4.8 μM MCCB4 (EC_50_) and harvested at the time points indicated. These data are shown as percentages of the DMSO only transfection control and demonstrated a statistically significant inhibition of luciferase from 12 h post transfection (hpt), with a 50% inhibition at 24 and 48 hpt (Fig. 3A). Between 0 and 6 hpt the luciferase signal was indistinguishable from background levels.

To further probe the potential of MCCB4 as a drug lead for the treatment of EBOV infection, the activity of MCCB4 on established NP-VP35 complexes was investigated as an indication of possible post exposure treatment. MCCB4 at a concentration equivalent to the EC_90_ (49 μM) was added to BSR-T7 cells at different time points pre- (−24–0 hpt), during (0–48 hpt & 0–24 hpt) and post-transfection (24–48 hpt) (Fig. 3B, top panel). Cells were harvested at 48 hpt and the data shown as a percentage of a DMSO only control. Pre-treatment of cells with MCCB4 followed by removal at transfection did not reduce the luciferase signal detected (Fig. 3B, −24–0 hpt). As expected, the addition of MCCB4 at transfection was able to disrupt EBOV replication complex formation, as demonstrated by the 85% reduction in luciferase (Fig. 3B and 0–48 hpt). The addition of MCCB4 post-transfection (Fig. 3B and 24–48 hpt) also reduced luciferase signal by 84%. This suggested that MCCB4 was able to effectively compete for binding to NP in established replication complexes. Interestingly, while the addition of MCCB4 at transfection and subsequent removal (Fig. 3B and 0–24 hpt) revealed a 65% reduction in luciferase compared to the DMSO only control – this reduction was significantly less than that displayed by both the sustained addition (0–48 hpt) and the post transfection addition (24–48 hpt).

### 3.3 MCCB4 activity is observed in two different cell types

**Fig. 4:**
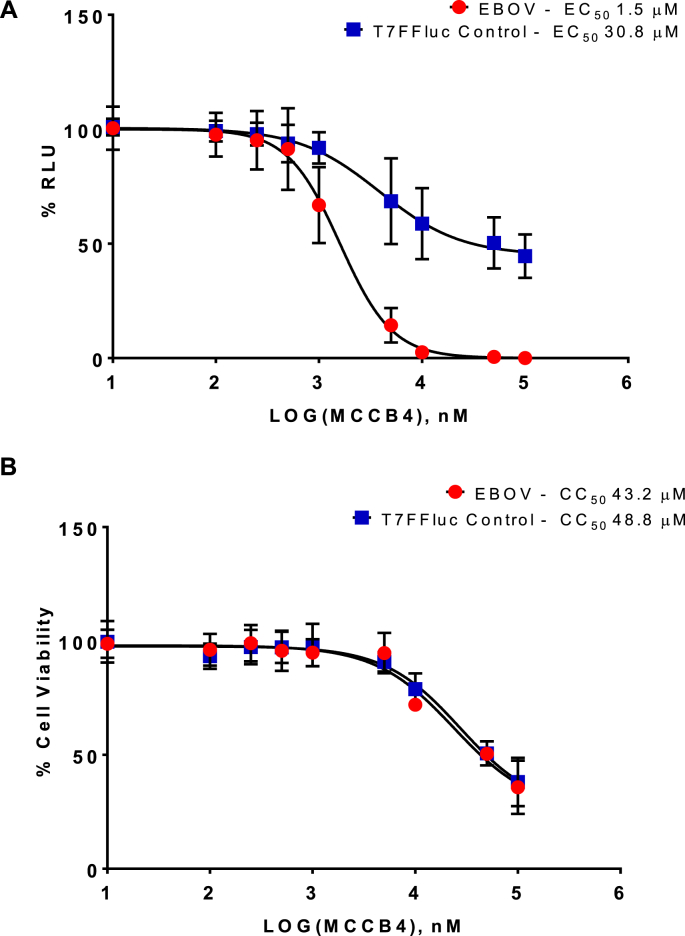
**MCCB4 activity in Huh7-Lunet-T7 cells.** MCCB4 was tested for effect against the T7FFLuc control, and EBOV minigenome in Huh7-Lunet-T7 cells. The compounds were added to cells at 100, 50, 10, 5, 1, 0.5, 0.25 0.1 and 0.01 μM immediately before transfection. Cells were harvested at 24 hpt and analysed for Firefly luciferase activity. Values are represented as percentages of a transfection only control. **A.** EC_50_ curve of percentage reduction in luciferase. EBOV, red circles. T7FFLuc control, blue squares. **B.** CC_50_ curve of percentage reduction in cell viability. EBOV, red circles. T7FFLuc control, blue squares. Averages plotted from 3 independent experiments performed in triplicate. Error bars show SD. (For interpretation of the references to colour in this figure legend, the reader is referred to the Web version of this article.)

To ensure that the effect of MCCB4 was not specific to BSR-T7 cells, EC_50_ and CC_50_ analyses were performed in Huh7-Lunet-T7 cells, a human hepatocellular carcinoma cell line which also expressed the T7 RNA polymerase. Both fibroblasts and hepatocytes are capable of supporting Ebola replication (Baskerville et al., 1985). The transfection efficacy of the EBOV MG and the T7FFLuc control in the different cell lines were similar (data not shown). In Huh7-Lunet-T7 cells, MCCB4 was shown to be effective with an EC_50_ 1.5 μM (Fig. 4A). MCCB4 exhibited a low level of cytotoxicity (CC_50_ 43.2 μM) and a low level of activity against the T7FFLuc control (EC_50_ 30.8 μM) in the Huh7-Lunet-T7 cells (Fig. 4B).

### 3.4 Validation of MCCB4-NP interaction through mutation of the NP hydrophobic pocket

**Fig. 5:**
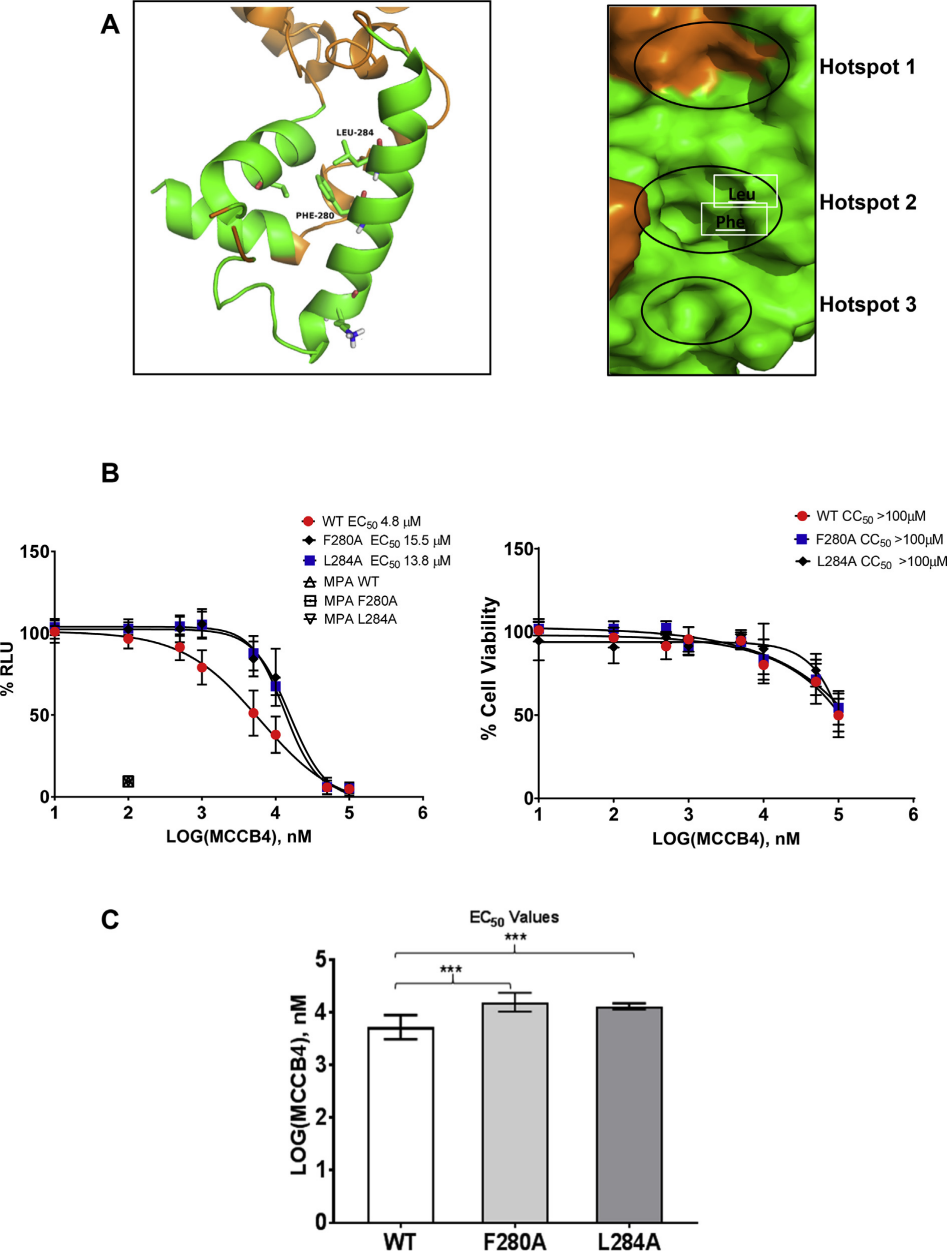
**Mutagenic analysis of NP confirms the mode of action of MCCB4. A.** The EBOV NP hotspot 2 residues Phe280 and Leu284 were mutated to alanine (F280A and L284A, respectively). **B.** EC_50_ curve of percentage reduction in luciferase. MCCB4 was added to cells at 100, 50, 10, 5, 1, 0.5, 0.1, and 0.01 μM immediately before transfection with either wildtype EBOV NP, mutant F280A or L284A. Cells were harvested at 24 hpt and analysed for Firefly luciferase. Values are represented as percentages of a transfection only control. **C.** Comparison of EC_50_ values generated from the wildtype NP or mutant NP transfections. EC_50_ values from 3 independent experiments performed in triplicate were plotted and compared using an unpaired two-tailed *t*-test. Error bars show SD. *** P Value < 0.001.

Although MCCB4 was predicted using eHiTs and AutoDock software to bind the EBOV NP, it was possible that MCCB4 acted on an alternative target. In order to validate the predicted targeting of MCCB4 to NP, we studied the effect of specific mutants of NP on the inhibitory activity of MCCB4. Specifically, residues F280 and L284 within NP, predicted to interact with MCCB4, were substituted with alanines (NP F280A and L284A) (Fig. 5A).

Cells were transfected with the EBOV minigenome plasmids including either wildtype or the 2 mutant NPs and treated with MCCB4 over a 10-fold range of concentrations. Of note, although the two mutations resulted in a 10-fold reduction in RLU compared to wildtype, they were replication competent (Supplementary Fig. 2). The low levels of RLU were not due to residual unspecific reporter gene activity as they were significantly higher than the values obtained in the absence of the plasmid expressing the L polymerase. These data were compared to the relevant transfection-only control and shown as a percentage of this to take into account any discrepancy between absolute units of luciferase. As a control, cells were treated with mycophenolic acid (MPA), an inosine monophosphate dehydrogenase inhibitor which inhibits RNA virus replication (Khan et al., 2011), including EBOV (Johansen et al., 2015). As expected, MPA at the EC_90_ concentration affected all three NPs equally.

While MCCB4 inhibited both EBOV NP mutants at high concentrations, the EC_50_ values were significantly higher than those shown by the wildtype NP (F280A 15.5 μM, L284A 13.8 μM Fig. 5B and C), demonstrating that these mutations resulted in partial resistance to MCCB4. MCCB4 cytotoxicity (CC_50_ > 100 μM, Fig. 5B) was not affected by the substitution of wildtype NP with either of the mutants. These data are consistent with the predicted binding of MCCB4 to the hydrophobic pocket within NP.

### 3.5 Structure-activity relationships of MCCB4 and related analogues

**Fig. 6:**
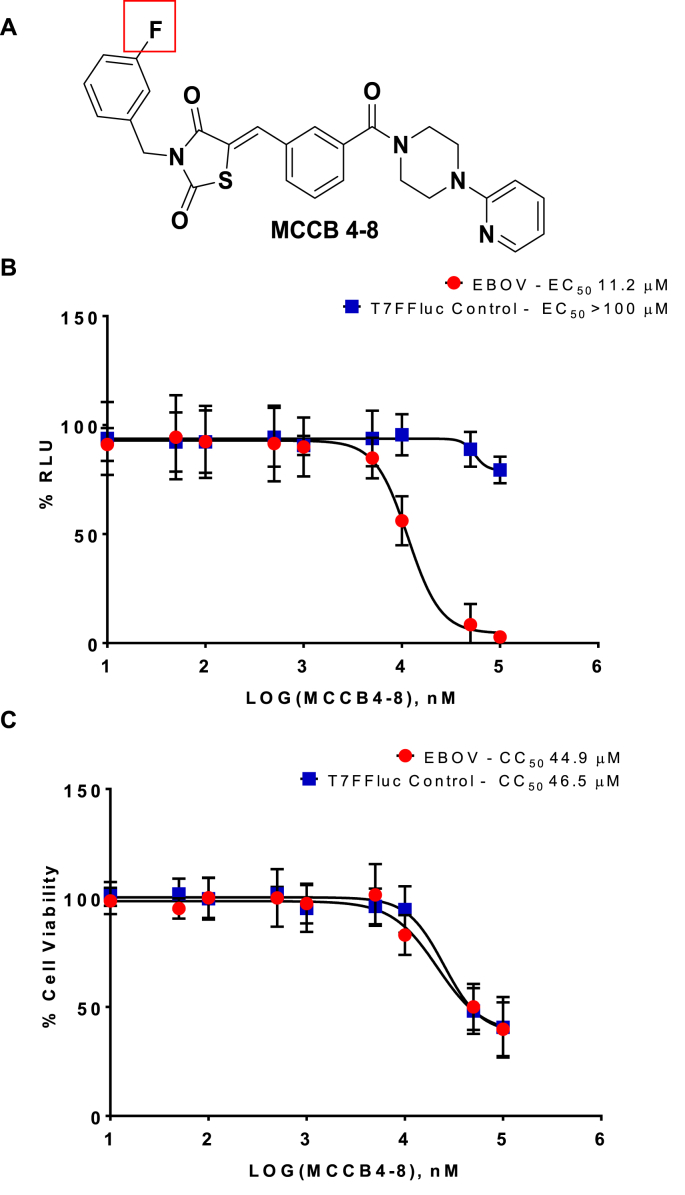
**SAR analysis of MCCB4-8.** The MCCB4 derivative MCCB4-8 was tested for effect against the T7FFLuc control, and EBOV minigenome. The compounds were added to BSR-T7 cells at 100, 50, 10, 5, 1, 0.5, 0.1, 0.05 and 0.01 μM immediately before transfection. Cells were harvested at 24 hpt and analysed for Firefly luciferase. Values are represented as percentages of a transfection only control. **A.** Structure of the MCCB4-8 compound. **B.** EC_50_ curve of percentage reduction in luciferase. EBOV, red circles. T7FFLuc control, blue squares. **C.** CC_50_ curve of percentage reduction in cell viability. EBOV, red circles. T7FFLuc control, blue squares. Averages plotted from 3 independent experiments performed in triplicate. Error bars show SD. (For interpretation of the references to colour in this figure legend, the reader is referred to the Web version of this article.)

**Fig. 7:**
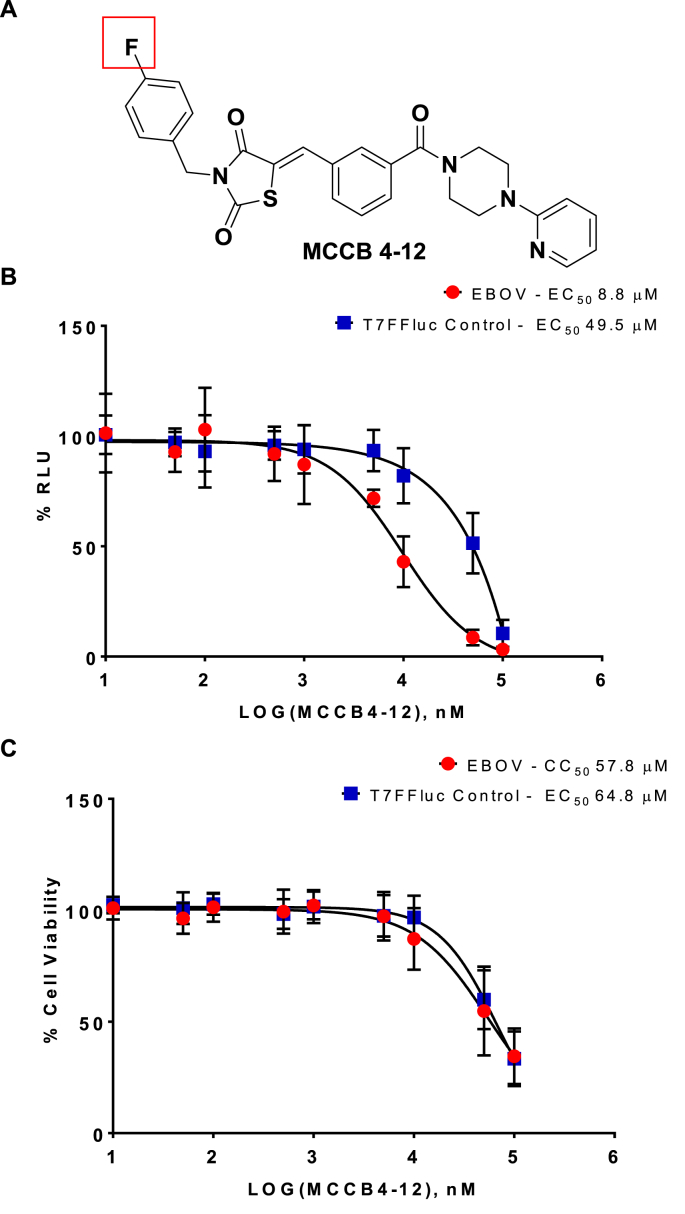
**SAR analysis of MCCB4-12.** The MCCB4 derivative MCCB4-12 was tested for effect against the T7FFLUC control, and EBOV minigenome. The compounds were added to BSR-T7 cells at 100, 50, 10, 5, 1, 0.5, 0.1, 0.05 and 0.01 μM immediately before transfection. Cells were harvested at 24 hpt and analysed for Firefly luciferase. Values are represented as percentages of a transfection only control. **A.** Structure of the MCCB4-12 compound. **B.** EC_50_ curve of percentage reduction in luciferase. EBOV, red circles. T7FFLuc control, blue squares. **C.** CC_50_ curve of percentage reduction in cell viability. EBOV, red circles. T7FFLuc control, blue squares. Averages plotted from 3 independent experiments performed in triplicate. Error bars show SD. (For interpretation of the references to colour in this figure legend, the reader is referred to the Web version of this article.)

In order to rapidly ascertain structure-activity relationship (SAR) data for MCCB4, five close structural derivatives, where particular functional groups had been modified, were purchased (from ChemDiv Inc) and a further eight ChemBridge analogues were identified using the programme ROCS (rapid overlay of chemical structures) (OpenEye Scientific Software, Santa Fe, NM. http://www.eyesopen.com) (Hawkins et al., 2007), a program which identifies compounds based on 3D shape similarity. Initially compounds were screened for efficacy compared to the MCCB4 parent compound. Two MCCB4 derivatives, MCCB4-8 (Fig. 6) and MCCB4-12 (Fig. 7) were identified as effective and so further investigated. Transfected cells were treated with the analogues over a range of concentrations (10 nM–100 μM). As shown in Fig. 6, Fig. 7, both MCCB4-8 and MCCB4-12 exhibited a dose-dependent inhibition of the EBOV MG.

MCCB4-8 is identical to the parent compound MCCB4 except for a fluorine at the 3-position of the terminal benzene ring (Fig. 6A, red box). MCCB4-8 has an EBOV specific EC_50_ 11.2 μM (Fig. 6B) and a CC_50_ of 45 μM (Fig. 6C EBOV). MCCB4-12 is identical to the parent compound MCCB4 except for a fluorine at the 4-position of the terminal benzene ring (Fig. 7A, red box). MCCB4-12 has an EBOV specific EC_50_ 8.8 μM (Fig. 7B) and a CC_50_ of ∼58 μM (Fig. 7C EBOV and T7FFLuc). The higher EC_50_ values and the lower CC_50_ values for these derivatives demonstrate that these structural changes are not advantageous.

## 4 Discussion

The data presented here outlines an *in silico* screen of SMIs which are predicted to bind in the hydrophobic pocket on the EBOV NP.

MCCB4 is predicted to bind to hotspots 1 and 2 within the hydrophobic pocket on the NP structure (Fig. 1). The benzyl group of MCCB4 fills the deep hydrophobic hotspot 2 with the pyridyl group located in hotspot 1. From our computational analysis, compounds predicted to bind to NP had structural features that allowed the molecule to traverse both these hotspots. MCCB4 contains five ring motifs and also an ene-thiazolidinedione group (which is structurally similar to the rhodamine class of promiscuous inhibitors (Baell and Walters, 2014), which potentially decrease its attractiveness for further drug development. Whilst we were mindful of the potential for promiscuous inhibition of additional cellular processes, we did not observe any additional toxic effects in our cellular assays compared with other compounds which were identified as non-selective and toxic. MCCB4 is present in screening collections but has not been reported in the scientific literature or in Chembl, the bioactive screening database.

MCCB4 was shown to be efficacious in reducing the luciferase signal detected in EBOV MG transfected BSR-T7 cells (EC_50_ 4.8 μM) and Huh7-Lunet-T7 cells (EC_50_ 1.5 μM). This effect was specific against EBOV replication as shown by high EC_50_ values (EC_50_ > 100 μM) demonstrated by both the T7FFLuc control transfection and the BUNV MG transfection. MCCB4 was well tolerated by cells, exhibiting a CC_50_ > 100 μM in BSR-T7 cells, although the compound appears to have a greater cytotoxicity in Huh7-Lunet-T7 cells (CC_50_ 43.2 μM). The reduction in luciferase signal presented by the T7FFLuc and BUNV MG control transfections tracks closely to cell viability so most likely reflects a reduction in the viability of transfected cells.

There are two distinct paths for potential EBOV treatments: post-exposure prophylaxis and treatment of symptomatic patients. Both have different challenges but a common strategy might be to limit virus replication to allow the adaptive and innate immune systems time to fight infection (Bray and Paragas, 2002; Feldmann et al., 2005). In this regard we demonstrated that MCCB4 can inhibit EBOV replication with a single dose at the time of transfection, but also was able to effectively inhibit replication when added at 24 hpt, supporting the possible use of MCCB4 as a post-exposure drug.

In order to confirm that MCCB4 interacts with EBOV NP at the targeted hydrophobic binding pocket, two alanine substitution mutants were generated (NP L_284_A and NP F_280_A) in this region. These changes were predicted to reduce binding efficiency. This pocket is the binding site of NP-VP35 during EBOV replication so a balance between retaining replicative function and disrupting the compound binding, must be met. As expected, the mutants exhibited a 10-fold reduction in replication (Supplementary Fig. 2). The mutations did not have any effect on the sensitivity to the non-specific antiviral compound MPA which was used as a control, nor the CC_50_ value of MCCB4 but did cause a significant EC_50_ shift between wildtype and mutant in response to MCCB4. This reduction was consistent with the hypothesis that MCCB4 binds to NP as predicted using *in silico* screening.

The selective index (SI = CC_50_/EC_50_) (Table 1) is a numerical method of comparing the therapeutic window of potential drugs, with a higher selective index being preferable. The EC_50_ values of the two MCCB4 analogues, MCCB4-8 and MCCB4-12, are higher than the parental compound (Table 1). In addition they exhibited lower CC_50_ values. Compounds MCCB4-8 and MCCB4-12, were predicted to enhance binding to EBOV NP via an enhanced hydrophobic effect, with the additional fluorine atom filling the deep hydrophobic hotspot 2. However, it is clear that the structural changes present in MCCB4-8 and MCCB4-12 adversely affected the efficacy of the compounds, pointing to a key role for the terminal benzene ring as a key player in the activity of MCCB4. This observation will inform future SAR that will focus on this part of the molecule in an attempt to improve efficacy.

**Table 1:** Summary of EC_50_ and CC_50_ data.

COMPOUND	TARGET	CELL	EC_50_	CC_50_	SI^a^
MCCB4	WT NP	BSR-T7	4.8 μM	>100 μM	>20.8
MCCB4	WT NP	Huh- Lunet-T7	1.5 μM	43.2 μM	28.8
MCCB4	NP F280A	BSR-T7	15.5 μM	>100 μM	>6.5
MCCB4	NP L284A	BSR-T7	13.8 μM	>100 μM	>7.2

MCCB4-8	WT NP	BSR-T7	11.2 μM	44.9 μM	4
MCCB4-12	WT NP	BSR-T7	8.8 μM	57.8 μM	6.5

a Selective Index (CC_50_/EC_50_).

## 5 Conclusions

Much research has been performed to investigate Ebola replication with a view to developing this knowledge into therapeutic strategies. Many SMIs have been tested *in vivo* and *in vitro* but this has yet to be translated into successful clinical studies and licenced drugs. Using the structure of NP, *in silico* screening was performed to identify SMIs including MCCB4. MCCB4 is efficacious, selective and well tolerated by cells. Limited SAR has been performed and it is our opinion that an extended investigation into the structure of MCCB4 could generate a superior compound suitable for *in vivo* investigations. As the NP protein is conserved and essential for replication, this drug discovery pipeline could generate a drug not only for EBOV but also related Filoviruses.

## Declarations of interest

None.

## Appendix A Supplementary data

Supplementary Fig 1. **The EBOV minigenome system. A.** Structure of EBOV support plasmids. **B.** Minigenome plasmid. The 5 plasmids are transfected into T7 polymerase expressing cells. Upon expression of L, NP, VP35 and VP30 the complex of these 4 proteins transcribe the minigenome to yield a translationally competent anti-genome that expresses Firefly luciferase.

Supplementary Fig 2. **A comparison of the activity of EBOV wildtype NP with the F280A and L284A mutants.** BSR-T7 cells were transfected with the EBOV MG plasmids: VP30, VP35, L and MG-FFluc; and either the wildtype NP, NP F280A or L284A. To assess non-specific reporter gene activity the L plasmid was omitted (- L). Cells were harvested at 24 hpt and analysed for Firefly luciferase expression. Experiments were performed in triplicate and compared using an unpaired two-tailed *t*-test. Values are represented as percentages of the complete plasmid complement for the wildtype NP. Error bars show SD. **** P < 0.0001, ** P < 0.01, * P < 0.1.
