# Supplementary material for: Identification of a small molecule inhibitor of Ebola virus genome replication and transcription using in silico screening
Source: Antiviral Res. 2018 Aug;156:46–54. doi: 10.1016/j.antiviral.2018.06.003 (PMC6371959; doi:10.1016/j.antiviral.2018.06.003)
Supplement: Multimedia component 1 [file mmc1.pptx]

## Slide 1
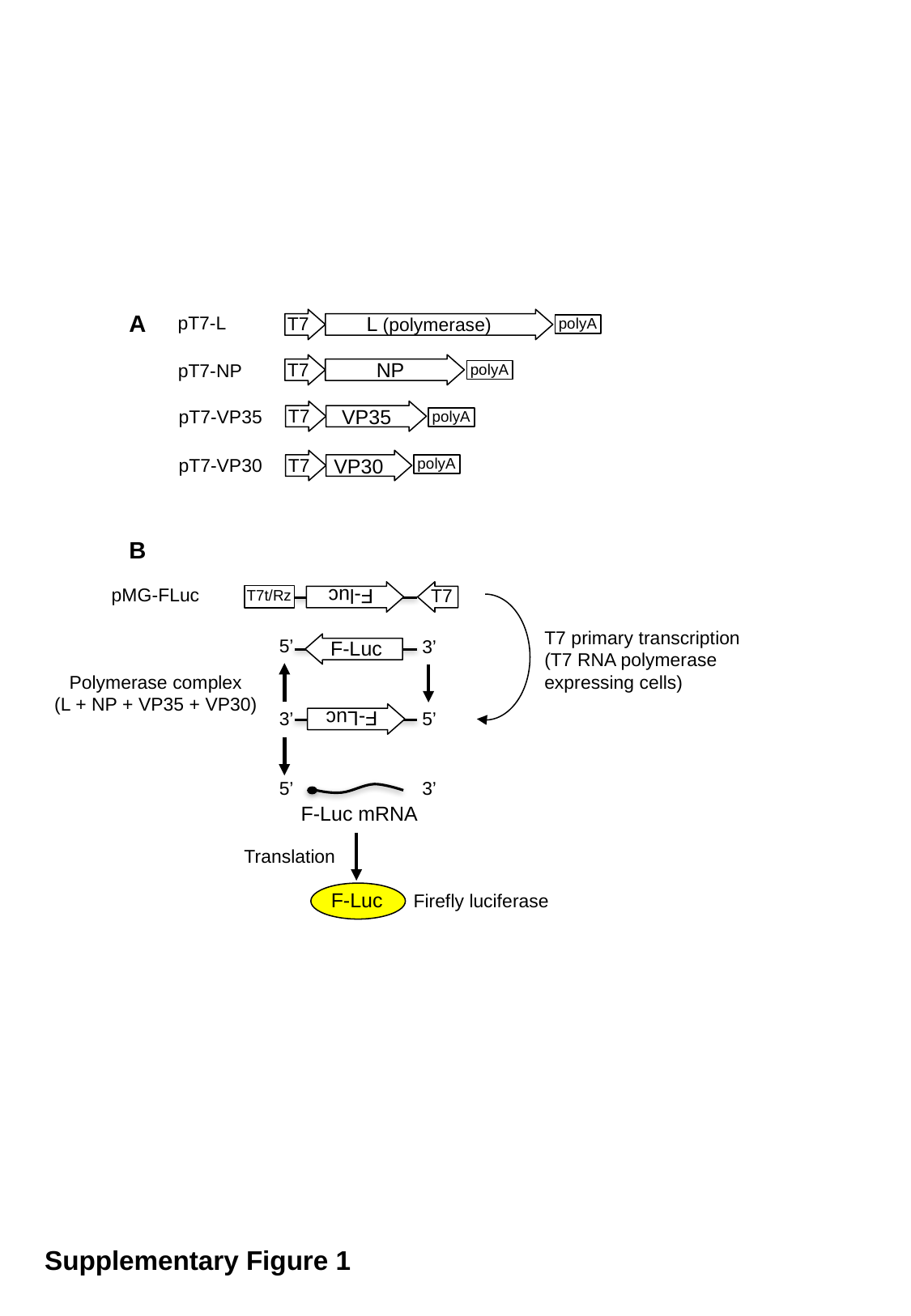

A
L (polymerase)
pT7-L
T7
polyA
NP
T7
pT7-NP
polyA
VP35
T7
pT7-VP35
polyA
VP30
pT7-VP30
T7
polyA
B
pMG-FLuc
T7
F-luc
T7t/Rz
T7 primary transcription
(T7 RNA polymerase
expressing cells)
5’
3’
F-Luc
Polymerase complex
(L + NP + VP35 + VP30)
3’
5’
F-Luc
5’
3’
F-Luc mRNA
Translation
F-Luc
Firefly luciferase
Supplementary Figure 1

## Slide 2
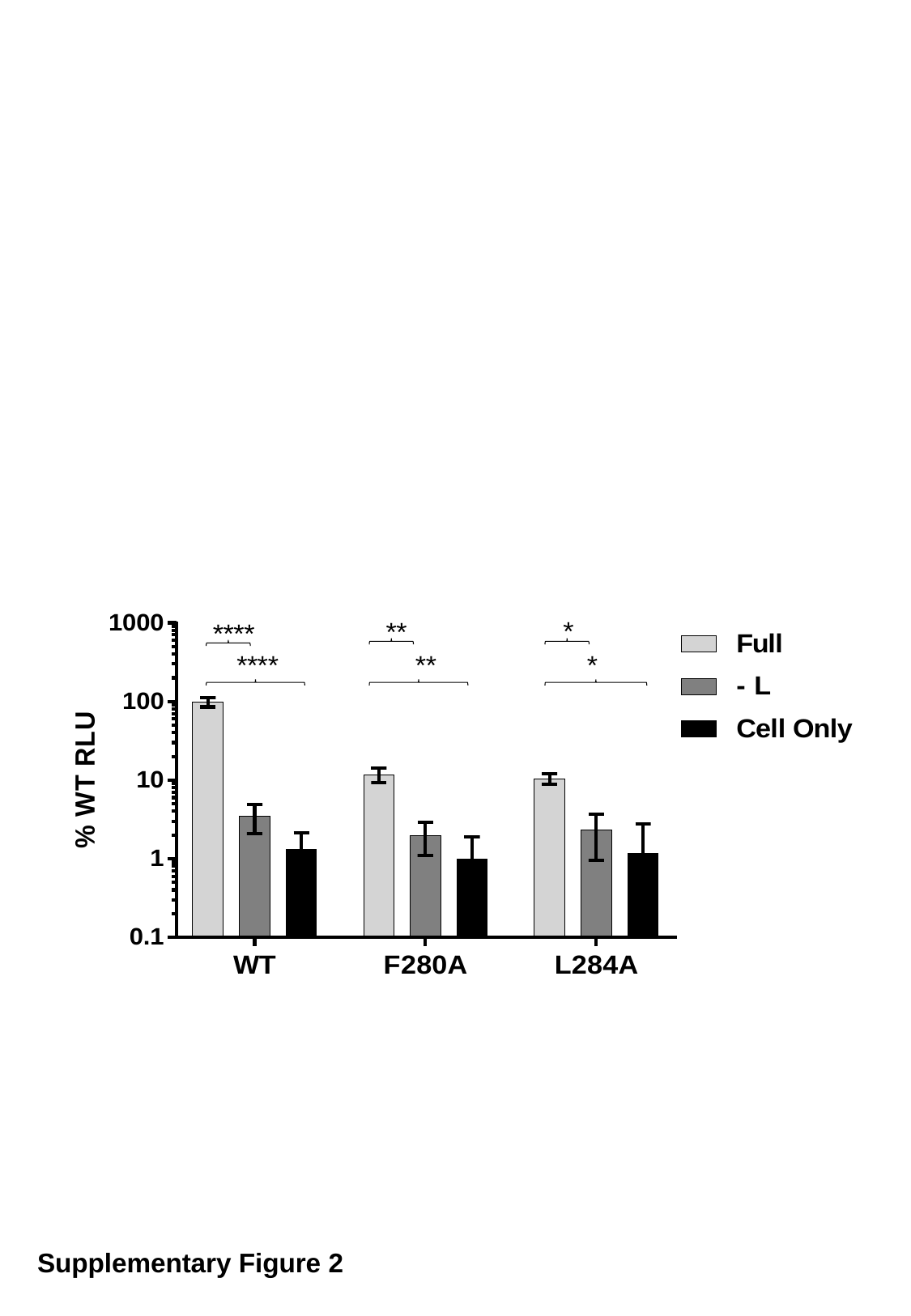

Supplementary Figure 2
